# Searching for New Beneficial Bacterial Isolates of Wild Raspberries for Biocontrol of Phytopathogens-Antagonistic Properties and Functional Characterization

**DOI:** 10.3390/ijms21249361

**Published:** 2020-12-08

**Authors:** Michał Pylak, Karolina Oszust, Magdalena Frąc

**Affiliations:** Institute of Agrophysics, Polish Academy of Sciences, Doświadczalna 4, 20-290 Lublin, Poland; m.pylak@ipan.lublin.pl (M.P.); k.oszust@ipan.lublin.pl (K.O.)

**Keywords:** beneficial bacteria, rhizosphere, metabolic analysis, phenotypic microarray

## Abstract

The threat caused by plants fungal and fungal-like pathogens is a serious problem in the organic farming of soft fruits. The European Commission regulations prohibit some commercially available chemical plant protection products, and instead recommend the use of natural methods for improving the microbial soil status and thus increasing resistance to biotic stresses caused by phytopathogens. The solution to this problem may be biopreparations based on, e.g., bacteria, especially those isolated from native local environments. To select proper bacterial candidates for biopreparation, research was provided to preliminarily ensure that those isolates are able not only to inhibit the growth of pathogens, but also to be metabolically effective. In the presented research sixty-five isolates were acquired and identified. Potentially pathogenic isolates were excluded from further research, and beneficial bacterial isolates were tested against the following plant pathogens: *Botrytis* spp., *Colletotrichum* spp., *Phytophthora* spp., and *Verticillium* spp. The eight most effective antagonists belonging to *Arthrobacter*, *Bacillus*, *Pseudomonas*, and *Rhodococcus* genera were subjected to metabolic and enzymatic analyses and a resistance to chemical stress survey, indicating to their potential as components of biopreparations for agroecology.

## 1. Introduction

Pathogenic microorganisms are a serious threat to crops, especially under the conditions in which the observed soil biodiversity is lowered. *Botrytis* spp., *Colletotrichum* spp., *Phytophthora* spp., and *Verticillium* spp. are just examples of the most symptomatic microorganisms that can cause plant diseases in the organic production of soft fruits including raspberries [1]. *Phytophthora* genus belongs to a common pathogens of raspberry and is one of the most crucial dangers in organic farming [2]. Research shows that representatives of the *Colletotrichum* genus can survive in soil and in any remaining plant residues for up to 30 months which might lead to re-infection even after defeating the disease in a particular growing season [3]. *Botrytis* genus is considered to be one of the most important plant fungal pathogens due to its potential to cause great losses in fruit yield [4].

The intensification of agricultural production, which has occurred in recent years, has led to farmers becoming dependent on chemical methods of plant protection as these methods are reliable and easy to use. In contrast to these advantages, chemical methods of plants protection may cause negative effects, i.e., the development of resistance by the targeted pathogen and a decrease in soil biodiversity [5].

Another disadvantage of conventional intensive cropping systems is the constantly growing cost of pesticides and the growing number of pathogens resistant to chemicals. The increase interest in organic farming and in particular, in the organic production of fruits could be considered as another factor encouraging farmers to develop new solutions in plant biostimulation and protection with the use of natural microbe-based products. European Commission regulation No. 834/2007 states that using chemical pesticides must be limited to the absolute minimum and farmers are encouraged to use substances of natural origin.

Furthermore, the EU Biodiversity Strategy for 2030, which was brought to life on 20 May 2020, sets forth the importance of biodiversity in all environments, and this includes increasing the biodiversity of agricultural land. This strategy for the coming years aims not only to reduce the use of pesticides by at least 50% but also to increase the area of organic farming until it reaches up to 25% of the total agricultural area. Organic farmers are obliged not to use many commercially available chemical pesticides and fertilizers. This can lead to lowering yields and an increase in the costs of this farming method. The aforementioned regulations also state that bioproducts, i.e., plant-based essential oils or biopreparations based on microorganisms are allowed and should be used in organic food production [6].

It was previously proven that deliberately selected native root-associated bacterial isolates, de novo introduced to cultivation, e.g., as biopreparations, can enhance plant growth [7]. In addition, they can rescue crops from diseases acting as biocontrol agents against a wide range of pathogens [8]. For this purpose, bacteria belonging to different genera, e.g., *Arthrobacter*, *Bacillus*, *Pseudomonas*, and *Rhodococcus* are frequently used [9,10,11].

Current research shows that locally isolated bacteria might be more effective against local pathogens than bacteria from different regions of the world [12]. Moreover, each plant species likely benefits from recruiting a specialized consortium of bacteria, which is specific for each plant system. Therefore, evidence of phytoprotective roles of microbes isolated from native environment should be evaluated under in vitro experiments and then in phytotron and agricultural conditions [8]. This indicates that it is important to collect new organisms from local sources and form new biopreparations based on them. Another factor is that bacteria isolated from wild raspberries growing in forests are less likely to have contact with pesticides which might decrease their antagonistic potential [13]. It also increases the survival chances of those organisms since they are still living in a similar ecological niche in terms of humidity or temperature.

Surely, the most expected outcome is that microorganisms introduced into the rhizosphere would present abilities to decompose organic matter, solubilize phosphorus, change soil structure creating small soil aggregates, and thus improve water retention. Beyond a doubt, the rhizosphere is a medium sufficient for bacterial growth., It contains organic acids, inorganic phosphorus, and organic carbon from rhizodeposition [14,15,16].

Nevertheless, the level of isolates survival and activity properties may be raised and influenced by different soil pH values or salinity levels in different ecological niches which they inhabit [10,11,12,13,14,15,16]. The very first and preliminary research suggesting prebiotic’ additives for the future application into microbial-based biopreparations was published [17].

As a consequence, these capabilities need to be taken into consideration during biopreparation construction and application. In novel probiotechnology actions, serving precision agriculture and targeted solutions, it seems to be reasonable to provide a wide range of descriptions of skills determinants. Among others, there is a potential to survive in different ecological niches, which we aimed to achieve the presented study. The objectives of the study were to acquire and widely characterize bacterial isolates from a native niche of raspberry plants in order to indicate its potential as components of future biotization and naturalization biopreparations for agroecology.

Referring to the above touchstones, the hypotheses of our study include (i) wild raspberry rhizosphere and the root are a valuable sources of new beneficial bacterial isolates, (ii) some of the isolates show a great but varied potential to inhibit the growth of *Botrytis*, *Colletotrichum*, *Phytophthora*, and/or *Verticillium* genera representatives, and (iii) based on metabolic abilities, enzymatic properties and resistance to chemical stresses of beneficial bacterial isolates and phytopathogens, it is possible to select the most probable additives for future biopreparations.

## 2. Results

### 2.1. Identification of Bacterial Isolates

**Figure 1:**
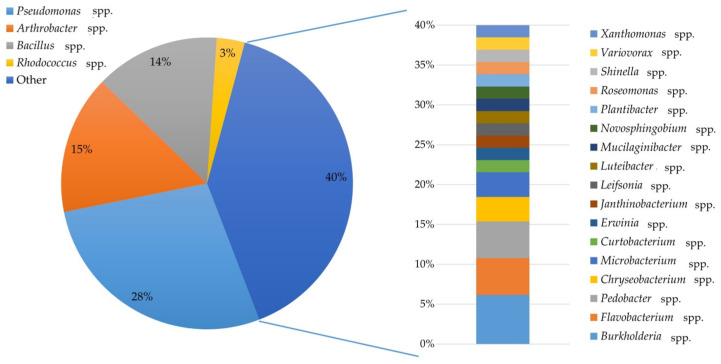
The percentage of bacteria belonging to individual genera among the isolates acquired from wild raspberry following identification based on 16S rDNA gene.

In the very first step, 65 different bacterial isolates belonging to 21 genera were isolated from wild raspberries and identified based on 16S rDNA fragment. The share of identified species is presented in Figure 1. Nine of the isolated bacteria were described as plant pathogens, and 42 isolates were recognized as potentially beneficial for plants. Among others, these were representatives belonging to the following genera *Arthrobacter* (15%), *Bacillus* (14%), *Pseudomonas* (28%), and *Rhodococcus* (3%).

**Figure 2:**
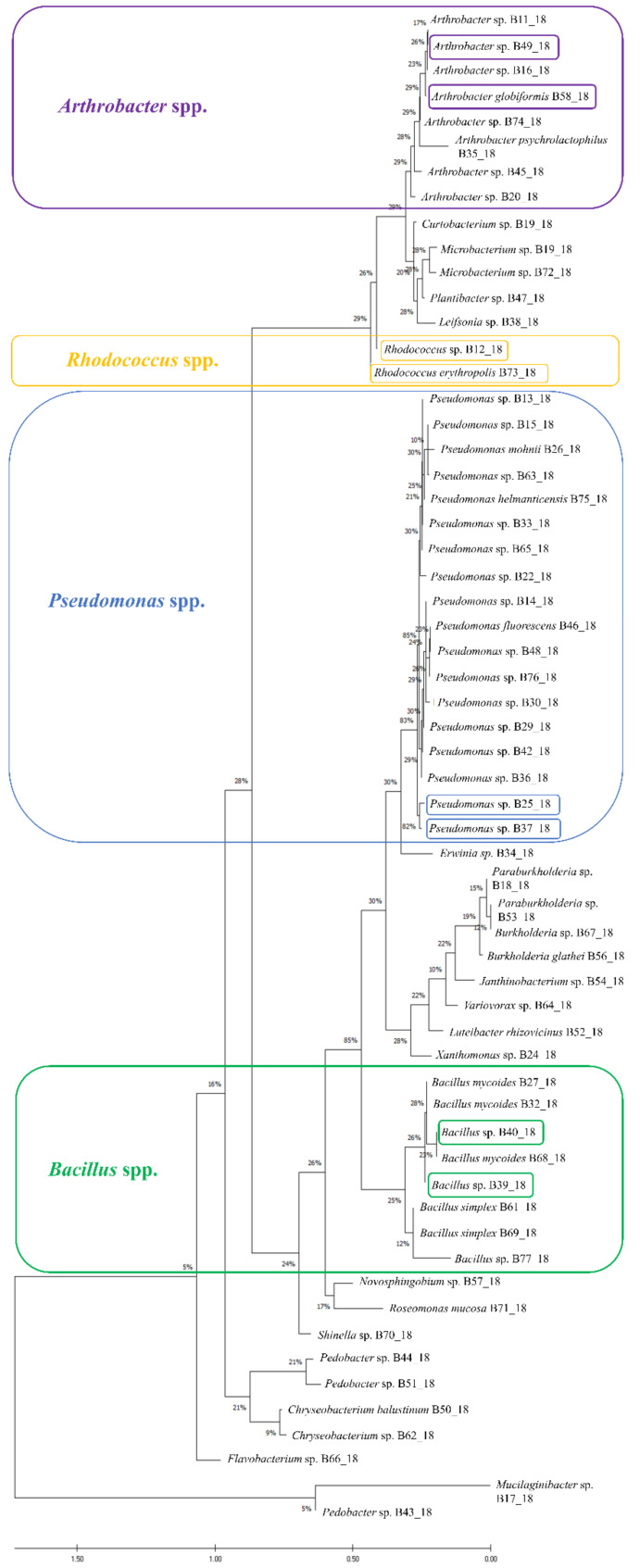
The evolutionary history of bacterial isolates of wild raspberry inferred by using the Maximum Likelihood method and Tamura-Nei model and bootstrap method based on 500 replicates. The tree with the highest log likelihood (−11,216.66) is shown. The percentage of trees in which the associated taxa clustered together is shown next to the branches. Initial tree for the heuristic search were obtained automatically by applying Neighbor-Join and BioNJ algorithms to a matrix of pairwise distances estimated using the Tamura-Nei model, and then selecting the topology with superior log likelihood value. The proportion of sites where at least 1 unambiguous base is present in at least 1 sequence for each descendent clade is shown next to each internal node in the tree. This analysis involved 60 nucleotide sequences. There were a total of 1629 positions in the final dataset. Evolutionary analyses were conducted in MEGA X. Colored brackets represent the main genera used in this research, and small brackets point to particular isolates tested in presented experiments.

Based on sequences obtained via the sequencing of the DNA fragment of 16S ribosomal DNA (rDNA) a phylogenetic tree was created including all identified isolates (Figure 2). All genera were easily distinguishable from each other and create their separate clusters. Genetic analysis revealed that there are two clusters among isolates belonging to the *Pseudomonas* genus. It is worth noting that both *Pseudomonas* sp. that were chosen based on their antagonistic properties belonged to only one small group. This suggests that they are closely related isolates.

### 2.2. Antagonistic Properties

The second step of our study covered isolates screening for their antagonistic abilities against the fungal and fungal-like pathogens. The results of these analyses are presented in Appendix A Table A1 and most promising isolates are summarized in Table 1. The list of tested isolates was narrowed in this step to the potentially beneficial ones. Among this, there were eight isolates of bacteria found to be characterized by relatively high degree of efficiency in inhibiting pathogens growth, which had no antagonistic properties to each other (data are not shown), this ensures that they will not interact in any negative way. These were the following isolates: *Arthrobacter* sp. B58/18 and B49/18, *Pseudomonas* sp. B25/18 and B37/18, *Bacillus* B39/18 and B40/18, and *Rhodococcus* sp. B12/18 and B73/18. The three bacterial isolates were the most effective in inhibiting the growth of pathogens. *Arthrobacter* sp. B58/18, *Pseudomonas* sp. B25/18 and *Rhodococcus* sp. B12/18 presented strong antagonistic properties against tested isolates from both *Colletotrichum* spp. Almost all of the tested bacteria were successful against *Phytophthora* spp. and *Verticillium* spp. representatives. Five bacterial isolates were effective in inhibiting the growth of *Botrytis* spp. and they were *Rhodococcus* sp. B12/18, *Bacillus* sp. B39/18, *Arthrobacter* sp. B 49/18 and B58/18, as well as *Rhodococcus* sp. B73/18.

The antagonistic properties varied among the examined bacterial isolates and different pathogens, nevertheless all bacteria except *Bacillus* sp. B40/18 were effective in inhibiting the growth of at least 7 strains of plant pathogens belonging to at least 2 genera. *Rhodococcus* sp. B12/18 influenced the growth of 11 different strains of pathogens and isolate *Bacillus* sp. B40/18 affected the growth of only 6. It is noteworthy that the bacteria examined did not cause a strong inhibition of fungal pathogens growth on plates but sometimes caused the inhibition of sporulation, e.g., *Arthrobacter* sp. B58/18 against *Botrytis* spp. G277/18. Appendix A Table A1 presents extended table of antagonisms against plants fungal pathogens of all isolated and potentially beneficial bacterial isolates.

### 2.3. Analysis of Metabolic Abilities

**Figure 3:**
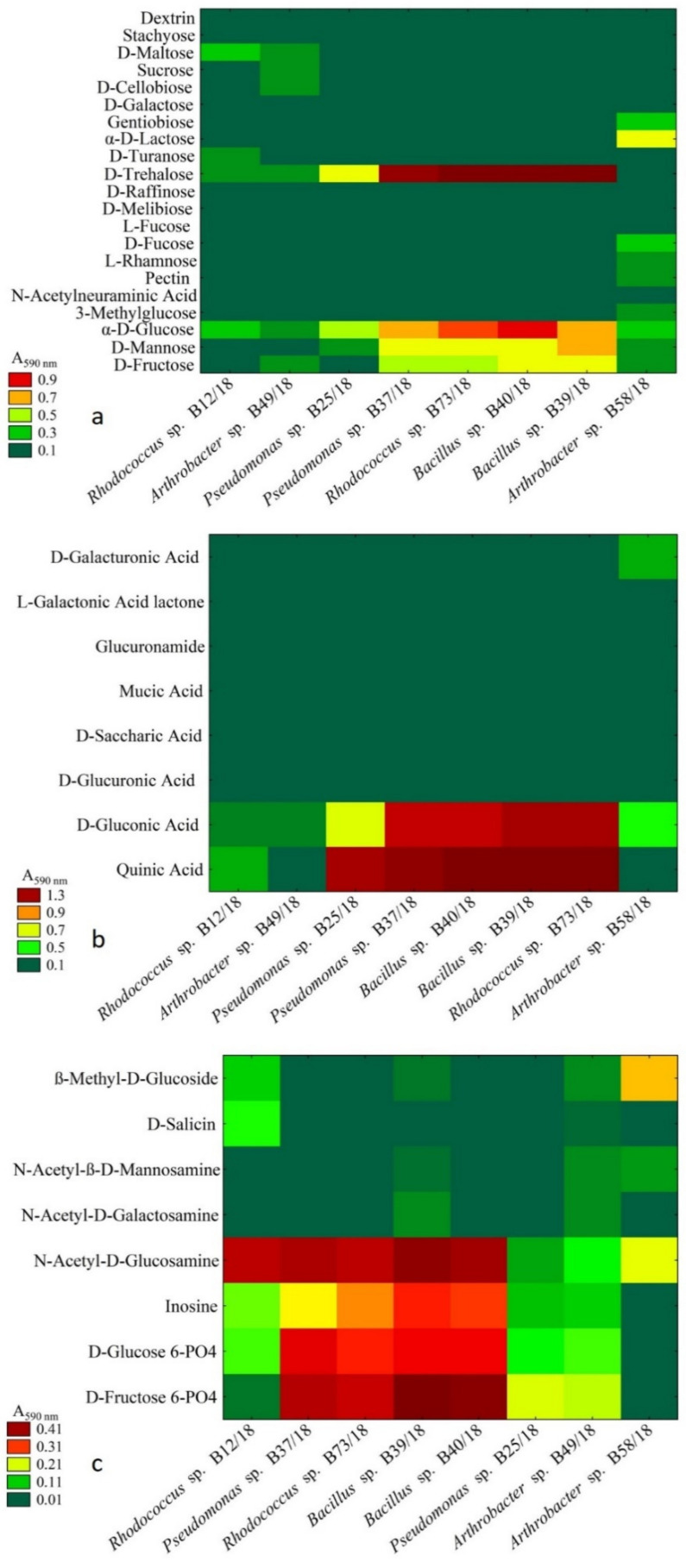
Cluster analysis of the examined bacterial isolates which depended on the utilization of carbon substrates–sugars (**a**), sugar acids (**b**), sugar derivatives (**c**). Measurements were taken for the wavelength of 590 nm, *n* = 3.

Following great antagonistic abilities of isolates *Arthrobacter* sp. B58/18 and B49/18, *Pseudomonas* sp. B25/18 and B37/18, *Bacillus* B39/18 and B40/18, and *Rhodococcus* sp. B12/18 and B73/18, they were next surveyed with metabolic abilities, namely carbon substrates—sugar, sugar derivatives, sugar acids (Figure 3a–c respectively).

Sugars are known to be the most commonly used carbon sources in growing substrates for bacteria. Even though sugars are the broadest group in this study only a few of them were attainable for the examined bacteria. In this study the most utilized carbon sources were α-D-glucose and D-trehalose followed by D-mannose and D-fructose. D-glucose was also the only compound utilized by every bacterium in this study, and *Arthrobacter* sp. B58/18 was able to make use of the broadest range of substances as a carbon source.

**Figure 4:**
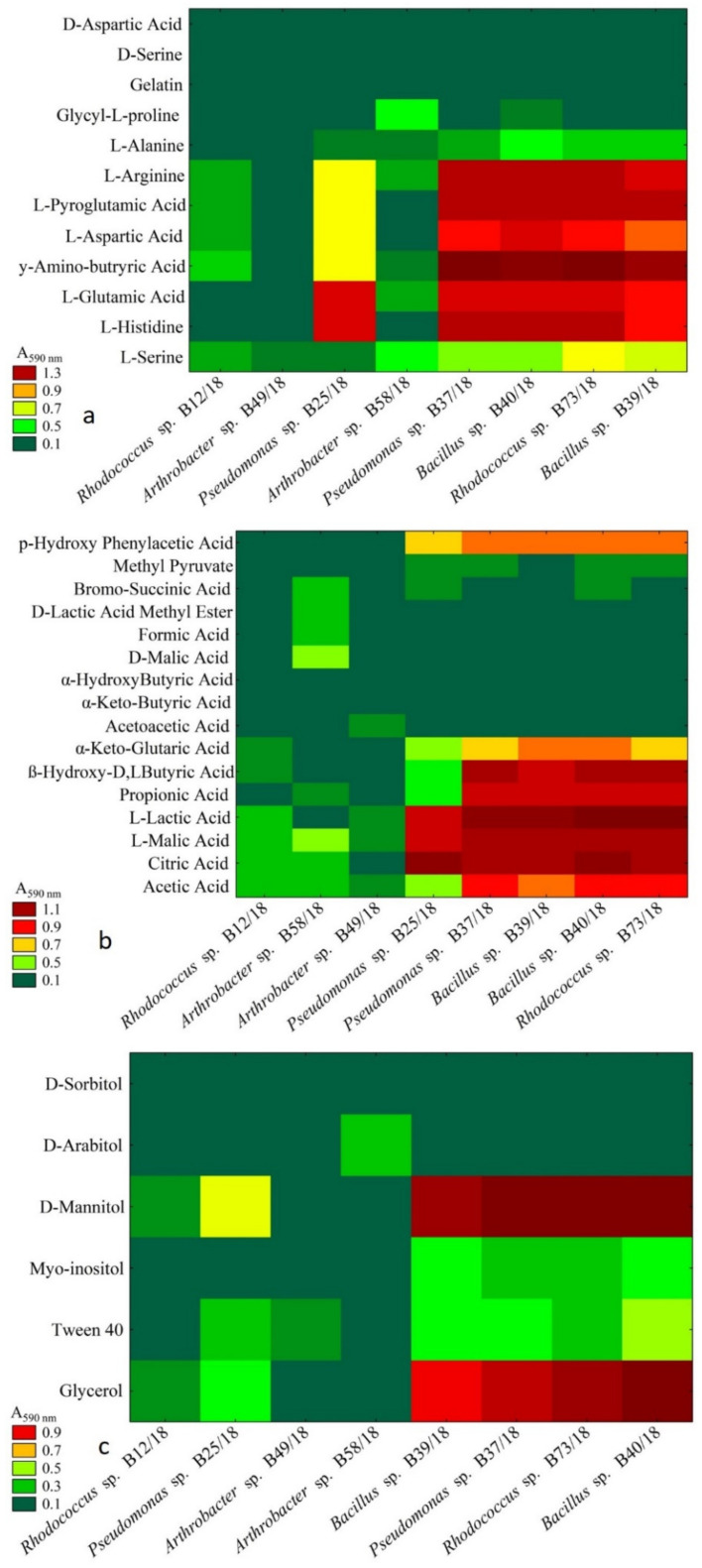
Cluster analysis of the examined bacterial isolates depended on the utilization of carbon substrates–amino acids, peptides and polypeptides (**a**), carboxylic acids and esters (**b**), polyols and others (**c**). Measurements were taken at a wavelength of 590 nm, *n* = 3.

It has been established that some sugar derivatives can be used as a carbon source in microbial growth or in biotransformation [18]. In this case, four compounds were utilized the most, and the result of the cluster analysis is presented in Figure 3 and these are as follows: D-fructose 6-phosphate and D-glucose 6-phosphate, inosine and N-acetyl. Only four bacteria were able to fully exploit these four substrates *Pseudomonas* sp. (B37/18), *Rhodococcus* sp. (B73/18), and *Bacillus* spp. (B40/18 and B39/18). Other substrates were not utilized in a significant way and it is noteworthy that *Arthrobacter* sp. (B58/18) was able to utilize only three of the sugar derivatives presented. Among many chemical compounds belonging to the group of sugar acids and their compounds, quinic acid was the one that was utilized by most of the isolates (Figure 4). D-gluconic acid was the second most utilized compound in this group. No other compounds were utilized at all.

As presented in Figure 4, every substrate except gelatin, D-serine, and D-aspartic acid was utilized. The only substrate that was utilized by every examined bacterium was L-serine. L-arginine, L-pyroglutamic acid, L-aspartic acid, L-glutamic acid, and L-histidine were utilized most profoundly by five isolates *Pseudomonas* spp. B25/18 and B37/18, *Bacillus* spp. B40/18 and B39/18, and *Rhodococcus* sp. B73/18. Similar results were obtained when checking the OD using the wavelength of 750 nm. The ratio between both absorbances for almost every isolate was above 1, which suggests a stressful metabolic situation (Table 2).

The carboxylic acids shown in the presented graph (Figure 4) are also known for being a potential carbon source for microorganisms [19]. In this case, the ones that were the most utilized by most isolates were acetic acid, citric acid, L-malic acid, L-lactic acid, propionic acid, β*-*hydroxy-D, L-butyric acid. It is worth noting that L-malic acid was the only compound that was utilized by every examined isolate. Isolates such as the following: *Rhodococcus* sp. B12/18, *Arthrobacter* spp. B58/18, and B49/18*,* were able to utilize the smallest number of compounds. The highest absorbance ratio values were presented for *Arthrobacter* sp. B58/18 (4.76)*,* the values for the other isolates varied from 0.84 to 2.31.

It has been proven that for some microorganisms polyols can serve an important role as carbon sources for their metabolism [20]. The polyols used in this study were glycerol and D-mannitol (Figure 4). *Arthrobacter* sp. B58/18 was able to utilize only one substrate D-arabitol, while D-sorbitol was not utilized by any isolate at all. Only two isolates were not able to grow on Tween 40 as the main carbon source and these were *Rhodococcus* sp. B12/18 and *Arthrobacter* sp. B58/18. Similar to the results of carboxylic acids and esters group utilization, the highest absorbance ratio values were demonstrated by *Arthrobacter* sp. B58/18 for sugars group as well as sugar acids and their compounds indicating the stressful metabolic situation, whereas the value for the other isolates varied from 0.00 to 0.85, which indicates a favourable utilization of substrate and a balance between respiration and biomass production.

**Figure 5:**
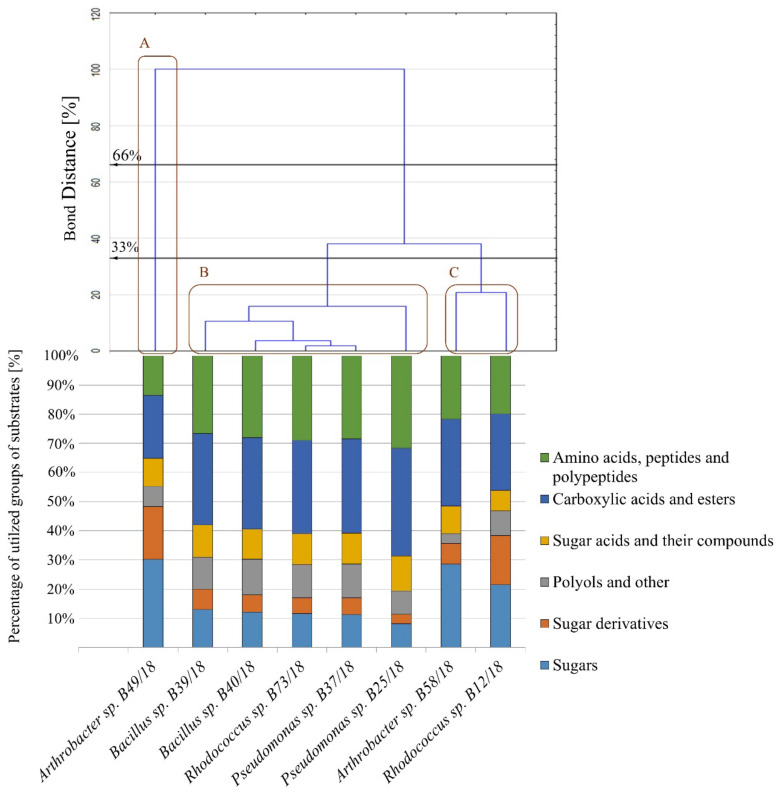
Bar graph presenting the ability to utilize different groups of substrates by the examined bacteria. The dendrogram presents a similarity in carbon sources utilization, clustering according to Sneath’s stringent criterion (33%) and the less restrictive criterion (66%). Utilization is calculated based on absorbance values at 590 nm, *n* = 3.

As the graph in Figure 5 shows, all bacteria except *Arthrobacter* sp. B49/18 were similar in their abilities to utilize particular groups of carbon sources. It barely utilized any compounds belonging to the following groups: amino acids, peptides and polypeptides, sugar acids and their compounds, and polyols and others. Instead, it was much better at utilizing sugars and sugar derivatives. Results for the other examined isolates were comparable. When considering Sneath’s stringent criterion (33%) there are three similar groups which may be distinguished (A–C). Regarding a less restrictive criterion (66%), the number of similar groups is only two.

### 2.4. Resistance to Chemical Stresses

Resistance to various factors, that may be encountered in a variety of environments is one of the most important features that characterize microorganisms intended to be used in biopreparations. Many factors influence the growth of microorganisms, and as it is widely known that a lack of carbon is one of the most significant [21]. As another factor, soil pH may be considered. Most European soils can be considered acidic as they have a pH value between 4 and 6. Over 21% of agricultural land in Poland is acidic (pH below 5.5) and this is a common problem for farmers [22,23].

Also, soil salinity may be a limiting factor in microbial growth [24]. As previously mentioned, during this study, absorbances were measured at two different wavelengths. The reason for this was that the absorbances for those wavelengths depend on different factors: 750 nm represents optical density, and 590 nm represents substrate usage. While presenting the results for different carbon source utilization we focused on the absorbances measured for 590 nm. In presenting the results of chemical resistance, we focused on the absorbance measured at 750 nm which represents optical density (OD) directly and is directly correlated with the number of bacterial cells in the growing medium.

**Figure 6:**
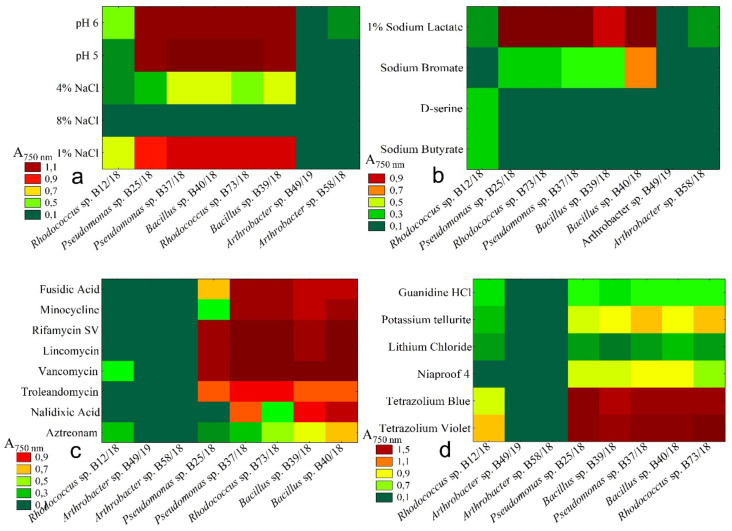
Charts presenting the medium absorbance values measured at 750 nm wavelength for different additives representing chemical stresses. The 4 charts represent the 4 groups of substrates: (**a**) chemical soil properties; (**b**) organic compounds, (**c**) antibiotics, (**d**) toxic substances.

As the data presented in Figure 6 show, the majority of the examined bacteria (*Pseudomonas* sp. B25/18, *Pseudomonas* sp. B37/18, *Rhodococcus* sp. B73/18, *Bacillus* sp. B39/18, *Bacillus* sp. B40/18) were resistant to most of the limiting factors with the exception of 8% NaCl, D-serine, and sodium bromate. They were able to grow almost in each given condition including the acidic pH (pH = 5), different salinity levels (1% and 4% NaCl). Some of tested isolates were also able to survive in the presence of selected antibiotics such as vancomycin, lincomycin, rifamycin, minocycline, fusidic acid, aztreonam, nalidixic acid and troleandomycin. Three of the examined isolates, i.e., *Rhodococcus* sp. (B12/18) and *Arthrobacter* spp. (B49/18 and B58/18), were very vulnerable to the conditions presented and barely able to grow at all. Nalixidic acid was also able to inhibit the growth of *Pseudomonas* sp. B25/18. 5 compounds were also effective in limiting the growth of all examined bacteria and these were 4% NaCl, sodium butyrate, guanidine hydrochloride, and lithium.

### 2.5. Enzymatic Activity of Selected Bacterial Isolates

The enzymatic activity of the three selected isolates were evaluated using both API ZYM tests (bioMérieux SA, Marcy l’Etoile, France) and Petri plates with media with appropriate substrates. The API tests allowed for the evaluation of the ability of bacteria to secrete 19 different enzymes and Petri tests allowed for eight more enzymatic abilities to be checked. The results are presented in Table 3.

The bacteria were different in terms of their metabolic and enzymatic abilities. Some enzymatic and metabolic abilities were greatly enhanced after a longer period of time such as the proteolytic abilities of *Pseudomonas* sp. B37/18. Although isolate B37/18 presented mediocre enzymatic abilities using API ZYM tests it was much more effective than the others when considering metabolic abilities. *Rhodococcus* sp. B12/18 presented limited metabolic potential with the capacity to conduct ammonification, denitrification and it only has slight cellulolytic abilities. However, B12/18 presented a broad spectrum of enzymatic abilities, being able to produce 11 different enzymes. *Arthrobacter* sp. B58/18 also secreted 11 enzymes used to evaluate enzymatic ability through API ZYM tests, but the overall activity was lower than that for *Rhodococcus* sp. B12/18.

### 2.6. Prebiotic Supplement Mixture against Fungal and Fungal-Like Pathogens

Based on the calculated ratio for both beneficial bacterial isolates and plant fungal pathogens, it was possible to note that some chemical compounds might be favourable as an addition to bacterial biopreparations, enhancing beneficial bacteria and influencing against plant fungal and fungal-like pathogens. The ratio results for all of the tested compounds are presented in Table A2. Those compounds are D-malic acid, D-saccharic acid, N-acetylo-D-galactosamine, and α-keto-glutaric acid. The results of our study indicated that they not only stimulate the growth of beneficial bacteria, but also, at the same time, cause a stressful metabolic situation for plant pathogens.

## 3. Discussion

The antagonistic properties of bacteria against fungal and fungal-like plant pathogens have been described previously, and efforts have been made to utilize these organisms in biological plant protection [25,26,27]. However, to the best of our knowledge, the metabolic characteristics of the presented organisms complied to a limited extent with their antagonistic properties against certain plant fungal pathogens, as proposed and determined in this research.

### 3.1. The Properties of Bacteria Belonging to Arthrobacter Genus

*Arthrobacter* sp. the isolate B58/18 was able to inhibit growth of selected phytopathogens and in this study was characterized by the ability to utilize a few carbon sources. Generally, the *Arthrobacter* genus representatives are known to produce amylase, lipase and protease [28]. Furthermore, *Arthrobacter* spp. can inhibit the growth of *Fusarium roseum* by releasing chitinase [29]. The ability to produce amylase may be useful in the utilization of compounds such as gentiobiose, pectin or other polysaccharides. In contrast, the production of protease can influence the utilization of glycyl-L-proline.

This particular isolate was also effective in the utilization of D-malic acid which may lead to the formation of acetic acid [30]. The previously mentioned abilities to produce chitinolytic enzymes may be a contributing factor to its noteworthy antagonistic properties. *Arthrobacter* spp. were successful in inhibiting the growth of the almost every isolate of chosen pathogens, especially isolates belonging to *Colletotrichum* spp. and *Phytophthora* spp.

It was established that *Arthrobacter* sp. B58/18 was susceptible to environmental stresses such as the presence of NaCl or antibiotics. However, this isolate does not pose a risk of introducing antibiotic-resistant organisms into the environment, which could cause unforeseen consequences for the environment and human. This result is important for further field application goals, when the formulated biopreparation based on this bacterial isolate is used for specific soil conditions. Thus, *Arthrobacter* sp. B58/18 is expected to be an effective antagonist, and the conditions for its proper development are predefined.

By contrast, *Arthrobacter* sp. B49/18 also is characterized by the lack of the ability to utilize a broad variety of carbon sources. However, compared to *Arthrobacter* sp. B58/18 which utilizes simple sugars such as D-fructose, D-mannose, and α-D-glucose, it is also similar in that it is vulnerable to environmental stresses. *Arthrobacter* sp. B49/18 proved itself to be less effective in inhibiting the growth of selected plant fungal and fungal-like pathogens than *Arthrobacter* sp. B58/18. These findings indicate that *Arthrobacter* sp. B49/18 is a rather weak candidate to be a constituent of a microbial biopreparation.

### 3.2. The Properties of Bacteria Belonging to Rhodococcus Genus

Furthermore, bacteria belonging to the *Rhodococcus* genus were also previously characterized in the literature as potentially beneficial microorganisms antagonistic to some plant pathogens. They were described to produce antimicrobial compounds such as the antifungals rhodopeptins, antimycobacterial lariatins or antibacterial aurachin RE, rhodostreptomycins A and B, saframycin A. These substances were proven to be successful in inhibiting the growth of many both bacterial and fungal plant pathogens which are common in agriculture [31,32,33,34].

In our study, we examined two isolates belonging to *Rhodococcus* genus that were isolated from wild raspberry roots and the rhizosphere. These were *Rhodococcus* sp. B73/18 and B12/18. Both isolates have been proven to be different from each other in their antagonistic properties against pathogens and their metabolic abilities and resistances to chemical stresses. The first one was successful in the growth inhibition of *Verticillium* spp. isolates. In turn, the second one inhibited the growth of *Phytophthora* spp. and two isolates of the *Botrytis* genera. Isolate B12/18 was overall better in its antagonistic properties inhibiting the growth of 11 isolates of phytopathogens. In terms of metabolic abilities and carbon source utilization, there is an easily visible contrast between those two isolates. Isolate B73/18 is much more effective in utilizing many different carbon sources than isolate B12/18. However, B73/18 isolate was resistant to many antibiotics. Therefore, due to the emergence of bacterial resistance to antibiotics, which is considered a worldwide public health problem, this isolate is a weaker candidate than B12/18 isolate as a biopreparation component.

However, the OD590/OD750 ratios were much lower for isolate B12/18 which might suggest that even though isolate B73/18 can utilize a broader spectrum of substrates it also triggers some stress-related mechanisms. OD590 increases during culturing but, OD750 does not increase accordingly. Although *Rhodococcus* sp. B12/18 was more vulnerable to different stress factors in comparison to isolate B73/18, it is the only bacteria in this study that was able to grow in the presence of D-serine and sodium bromate. Since developing a formulation of microorganisms for a biopreparation requires the step for satisfactory biomass production, this study includes a preliminary selection of media ingredients that may trigger biomass production with simultaneous relatively low level of metabolic reactivity. The metabolic response should not be very significant since this means that a rather stressful situation is in progress, as was explained in detail in recent years [17].

### 3.3. The Properties of Bacteria Belonging to Bacillus Genus

The bacteria belonging to the *Bacillus* genera are commonly used in many biopreparations [6]. They produce a wide array of antimicrobial compounds and have the ability to induce plant systemic resistance against pathogens such as *Botrytis cinerea* [35,36]. One of the most important substances produced by this genus of bacteria is lipopeptides which are highly antimicrobial [37]. Both of the tested *Bacillus* spp. isolates presented antagonistic properties against isolates of pathogens belonging to the *Verticillium* and *Phytophthora* genera. Even though *Bacillus* sp. isolate B39/18 was not producing a very strong antagonistic reaction to *Verticillium* spp., this isolate was not easily overgrown by these fungi and was able to compete for nutrients that might be vital to colonize soil and promote plants growth [38].

Furthermore, *Bacillus* sp. isolate B39/18 inhibited the growth of *Colletotrichum* sp. G166/18. Although some researchers have described the antagonistic properties of *Bacillus* sp. against *Colletotrichum* spp. results may vary depending on the particular isolates [39,40]. Isolate B39/18 was showing antagonistic properties against *Botrytis* spp. In terms of carbon sources utilization, both isolates were incredibly similar. It was only possible to observe small differences such as the broader spectrum of compounds utilized by *Bacillus* isolate B39/18, especially sugar derivatives. Although they are similar, the OD590/OD750 ratio suggests that *Bacillus* sp. isolate B40/18 handles stressful conditions better (ratios are almost the same or lower for this isolate).

### 3.4. The properties of Bacteria Belonging to Pseudomonas Genus

Last but not least, there are bacteria representatives belonging to the *Pseudomonas* genus used in commercially available bioproducts [6]. *Pseudomonas* strains were found to be effective in decreasing severity of red raspberry cane spur blight [41]. It has been proven that *Pseudomonas* spp. isolated from the rhizosphere of trees are able to inhibit the growth of some soil-borne plant pathogens e.g., *Verticillium* spp. [42]. *Phytophthora* spp. is also a pathogen that is inhibited by *Pseudomonas* spp. representatives. Research suggest that a treatment composed of *Pseudomonas fluorescens* and olive oil is more effective in reducing the severity of Phytophthora Blight of Pepper than acibenzolar-S-methyl (ASM) which is a newly introduced fungicide and systemic resistance stimulant [43,44].

In our research there were two isolates belonging to the *Pseudomonas* genus included. In terms of their antagonistic properties against fungal and fungal-like plant pathogens *Pseudomonas* sp. isolate B25/18 presented a very strong antagonistic mode against *Colletotrichum* spp., and the two isolates of *Phytophthora* genus. Isolate B25/18 was also effective in inhibiting the growth of *Verticillium* spp. In contrast, *Pseudomonas* sp. isolate B37/18 presented very weak antagonism against *Colletotrichum* spp. and similarly to B25/18, isolate B37/18 was found to inhibit the growth of *Verticillium* spp. and *Phytophthora* spp. (Table 1). Both isolates presented no antagonistic properties against *Botrytis* spp. Both isolates presented different carbon utilization patterns. Isolate B37/18 was more effective in terms of carbon utilization from different carbon sources. Isolate B25/18 grew less intensively on media containing amino acids, peptides, and polypeptides such as L-aspartic acid, L-pyroglutamic acid, L-arginine, and L-serine compared to isolate B37/18.

It has been proven that some bacteria belonging to the *Pseudomonas* genera can utilize the aforementioned amino acids as their sole source of carbon and nitrogen [45] which was also confirmed in the presented research. Both tested isolates, *Pseudomonas* sp. B25/18 and B37/18 were able to use carboxylic acids as a carbon source, although isolate B25/18 was less effective in utilizing acetic acid, propionic acid, β-hydroxy-D-butyric acid and α-keto-glutaric acid in comparison with the to isolate B37/18. The ability to utilize carboxylic acids by *Pseudomonas* spp. has previously been noted and corresponds with our results [46]. Myo-inositol was not utilized by *Pseudomonas* sp. isolate B25/18, as opposed to isolate B37/18, which was able to use it as a carbon source. Glycerol and D-mannitol which were not available for *Pseudomonas* sp. isolate B25/19, were utilized at a high rate by isolate B37/18. Glycerol has proved to be a sufficient carbon source for *Pseudomonas* spp. representatives. Bacteria cultured on a medium containing glycerol as the only carbon source tend to produce additional substances such as glycolipids [47]. Both isolates utilized the same 4 sugar derivatives: D-fructose 6-phosphate, D-fructose 6-phosphate, inosine, and N-acetyl-D-glucosamine, but the ability to utilize them was different. Isolate B37/18 was more effective in the utilization of those compounds. *Pseudomonas* sp. isolate B37/18 along with isolate B25/18 were able to grow on a medium containing one of three sugars: D-mannose, D-glucose, and D-trehalose. Moreover, isolate B37/18 grows on a medium containing D-fructose as the sole carbon source.

The chemical resistances of both isolates were similar but isolate B25/18 presented no resistance to nalidixic acid, and weaker resistance to potassium tellurite, 1% NaCl, fusidic acid, and minocycline compared to isolate B37/18. It has been proven that concentrations of NaCl higher than 1.75% reduce the amount of *Pseudomonas* bacteria growing in the medium [48]. Upon the initial review isolate B37/18 seemed to be much more effective in utilizing the presented compounds as carbon sources than isolate B25/18, but comparing these results to the OD590/OD750 ratio, it is visible that for isolate B37/18 the conditions presented were more stressful than for *Pseudomonas* sp. isolate B25/18. Therefore, while constructing biopreparations it should be taken into consideration that there are discrepancies between the two candidate isolates concerning antagonistic and substrate utilization properties, suggesting better potential of B37/18 isolate as a candidate for biotization and naturalization biopreparations.

### 3.5. Summary of Bacterial Properties as Potential Candidates to Biopreparations for Agroecology

However, it is worth noting that D-trehalose is utilized by the majority of the bacteria examined. Trehalose is a compound that can be used by bacteria as a carbon source but it is also produced by microorganisms while they are subjected to stressful conditions, especially on an osmotic basis [49]. It is also commonly used to increase the survivability rate when bacteria become too dry [50,51], which is an element of microorganism preservation. Those properties of trehalose make it an ingredient worth considering as an additive to bacterial biopreparations. It might not only increase the survivability of the bacterial formulation during storage, but it may also can enhance their growth in the natural environment after application.

Surprisingly, isolates such as *Pseudomonas* sp. B25/18 and B37/18, *Bacillus* sp. B39/18 and B40/18, *Rhodococcus* sp. B73/18 grew very effectively indeed in the presence of antibiotics. The release of antibiotics into soils creates a potential threat to microorganisms in this environment and may impact on the functional, genetic, and structural diversity of microbial communities, therefore probably some isolates acquired from soil environment pose such resistance. These compounds, if they occur in soil, are proven to affect its microbiota negatively, thereby decreasing soil microbial activity [52]. When choosing microorganisms to be used in biopreparation, especially for agroecology and organic production it might be important to select isolates that are not resistant to antibiotics. This will ensure a slower spread of antimicrobial properties against native soil inhabitants [53]. Since introducing antibiotic-resistant isolates to new ecological niches might have unforeseen consequences, it is needed to limit this type of organisms to a minimum and use them only when needed. Isolates B12/18 and B58/18 presented not only high metabolic activity, but also almost no resistance to antibiotics, making them good candidates for future biopreparation formulations.

The enzymatic and metabolic abilities may be the key to properly understanding the possible functions of those bacteria in the environment. The broader the spectrum of secreted enzymes the more environmental niches that bacteria can grow in and the more competitive they can be against other possibly pathogenic microorganisms [54]. The cellulolytic abilities presented by all of the examined isolates might contribute to an increase in the amount of organic matter in soil [55]. It is also worth noting that the addition to the soil of microorganisms that have a high degree of metabolic and enzymatic activity might stimulate different processes in the soil environment [56] and drive soil ecosystem services that are important in the soil quality evaluation [57]. Therefore, one of the strategies for agroecology could be the application of microbes used as a drivers of soil services [58] by participation in crucial processes in the soil environment through enzymatic activity of selected isolates and their consortia.

It is widely known that plants have their own probiotic bacteria similarly to animals. It is worth noting that when considering new formulations of biopreparations it is possible to enhance the further growth of microorganisms in particular environment by using certain additives [59]. It is also important for the additives not to enhance the growth of pathogens such as pathogenic fungi, especially if a biopreparation is to be used not only to stimulate the growth of plants, but mainly as an agent of plant fungal and fungal-like pathogen biocontrol.

The choice of the appropriate additives requires very specific research in order to evaluate their influence not only on probiotic microorganisms, but also on pathogens [60]. Table A2 presents different compounds whose influence on both beneficial bacterial isolates as well as fungal pathogens was tested in presented study. This approach allows one to perform a rapid and cost-effective evaluation over 40 different compounds. D-malic acid, D-saccharic acid, N-acetylo-D-galactosamine, and α-keto-glutaric acid that were indicated to be the best choice as prebiotic supplementary blend were not only easy to obtain, but also economical. Based on the OD590/OD750 and OD490/OD750 results, they seem to be the best out of all compounds tested.

This research is essential for future work focused on making a biopreparation composed of microorganisms to be used in the organic farming of soft fruits. An advanced knowledge base concerning the abilities of various bacteria to utilize different carbon sources might be important in deciding the future composition of a bacterial growing media or carrier for microorganisms. A deeper knowledge concerning how bacteria deal with stress factors, such as different pH or salinity levels, may help in predicting if they will be able to survive in the biopreparation or in the environment.

## 4. Materials and Methods

### 4.1. Bacterial Isolates Acquisition and Identification

The bacterial isolates used in this study were derived from the wild raspberries rhizosphere and root samples derived from Janów Lubelski, Kraśnik, Łuków, Puławy, Siedlce, and Świdnik Forest Distincts. First raspberry plants with adhering soil were collected and initially prepared as described by Oszust and Frąc [61] and followed by bacterial isolation procedures (from rhizosphere and roots). For this one gram of rhizosphere soil or root, the sample was placed in 9 mL sterile water (10^−1^) and shaken for 10 min at room temperature. One (1.0) mL of this suspension was transferred into a 9 mL blank (10^−2^) and serially diluted up to 10^−6^. Then, 100 µL of each dilution from 10^−4^ to 10^−6^ series was added to Petri dishes with Plate Count Agar (PCA, BioMaxima, Lublin, Poland) or medium based on soil extract [62] and incubated at 25 °C for 48–72 h. The morphologically distinct bacterial colonies were isolated and subcultured on Potato Dextrose Agar medium (PDA, A&A Biotechnology, Gdynia, Poland). At this stage of research 65 different bacterial isolates were distinguished, 63 from wild raspberry rhizosphere and 2 isolates from its roots therein.

Then, bacterial isolates were identified based on genetic identification with using commercially available Applied Biosystems™ Kits based on universal primers included (Applied Biosystems by Thermo Fisher Scientific, Waltham, MA, USA) as described below. All analyses were performed according to the producer’s manual in the Veriti™ 96-Well Thermal Cycler (Applied Biosystems by Thermo Fisher Scientific, Waltham, MA, USA) as presented below.

In the first step, the genomic DNA was extracted from a single colony for each bacterial isolate using PrepMan Ultra reagent (Applied Biosystems by Thermo Fisher Scientific, Waltham, MA, USA). Then there were PCR reactions conducted to amplify 16S rDNA gene with FAST MicroSeq™ 500 16S rDNA PCR Kit (Applied Biosystems by Thermo Fisher Scientific, Waltham, MA, USA). Thermal cycling conditions for this step were as follows: initial denaturation at 96 °C for 1 min, then 30 cycles of 15 s at 64 °C of annealing, and a final extension at 72 °C for 1 min.

Next, the quality of obtained DNA amplicons was assessed in 2% agarose gel in electrophoresis and followed by purification using ExoSAP-IT™ PCR Product Cleanup Reagent (Affymetrix Inc., Santa Clara, CA, USA) with the procedure that included incubation at 37 °C for 15 min and at 80 °C for 15 min was performed. Then, forward and reversed-sequencing reactions were prepared, and the cycle sequencing run was performed using MicroSEQ™ 500 16S rDNA Sequencing Kit (Life Technologies Ltd., Warrington, United Kingdom by Thermo Fisher Scientific, Waltham, MA, USA). The thermal cycling conditions for this step were as follows–initial denaturation at 96 for 1 min, 10 s melting at 96 °C, 50 °C, 5 s annealing in 25 cycles, and followed by 75 s extending at 60 °C. Subsequently, Performa Spin Columns (EdgeBio, San Jose, CA, USA) were applied to rid reagent residues. After that, the material was subjected to identification via Sanger sequencing [63] with Applied Biosystems 3130 sequencer (Applied Biosystems, Foster City, CA, USA).

In the next step, all obtained sequences were aligned and compared with the GenBank database (https://www.ncbi.nlm.nih.gov/genbank/). Subsequently, phylogenetic tree was prepared using the maximum likelihood method employing MEGA (University Park, PA, USA) software [64]. For this purpose, the Tamura–Nei model [65] and bootstrap method phylogeny testing were applied.

The detailed information about each bacterial isolate covering identification, accession number of sequences in GenBank (https://www.ncbi.nlm.nih.gov/genbank/), the sample from which it was isolated, the medium used for isolation, and the forest where samples were obtained with GPS-coordinates, are summarized in Table A3.

### 4.2. Biocontrol Efficacy and Antagonistic Abilities of Bacterial Isolates

Eight selected isolates, namely *Arthrobacter* spp. B49/18 and B58/18, *Bacillus* spp. B39/18 and B40/18, *Pseudomonas* spp. B25/18, B37/18, and *Rhodococcus* spp. B12/18 and B73/18, were tested for their antagonistic properties against 12 isolates of 4 selected plant fungal and fungal-like pathogens (three isolates of each pathogen): *Botrytis* spp., *Colletotrichum* spp., *Phytophthora* spp. and *Verticillium* spp. Phytopathogens characteristics including a way and source of isolation, as well as identification within GenBank accession numbers list were described previously by Malarczyk et al. [66]. The first step was to suspend centrifuged bacteria in 1 mL of water. After that, 90-mm diameter Petri dishes with 20 cm^3^ of PDA medium (A&A Biotechnology, Gdynia, Poland) were inoculated with 100 µL of homogenized pathogen mycelium by spreading pathogen inoculum (70% T) over the solid medium using sterile disposable spreaders. After inoculation, three 4 mm sterile cellulose paper circles were placed on the medium, at the same distance from each other. Then, 15 µL of bacterial suspension (90% T) was pipetted onto every circle and the plates were incubated at 25 °C for 96 h. The pathogens growth inhibition zones surrounding the circles with bacterial inoculum were measured after incubation.

The next step was to evaluate how the examined bacteria react to each other. This step ensures that it is possible to combine certain bacteria into one biopreparation. Any mutual antagonism would prevent those bacteria from being in one formulation. To do this, 90-mm Petri dishes, containing 20 cm^3^ of PDA medium (A&A Biotechnology, Gdynia, Poland) were inoculated with 100 µL of bacterial suspension (90% T). The bacterial inoculum was spread using sterile spreaders. After that 16 paper circles with a diameter of 4 mm were placed on the plates, in a pattern that guarantees the same distance from one paper circle to another. Next, 15 µL of bacterial suspension was pipetted onto every circle (90% T). The growth inhibition zone was measured after 96 h of incubation at 25 °C.

### 4.3. Metabolic Abilites of Isolated Beneficial Bacterial Isolates

The eight selected bacteria were tested on GEN III plates (Biolog Inc., Hayward, CA, USA) due to their very diverse and noteworthy substrate composition. One plate allows the researcher to check bacterial growth both on many carbon sources and in different stress conditions. There were 71 different carbon sources in a separate well of the plate, and we divided them, for further analysis, into 6 different groups based on their chemical structure: amino acids, peptides, and polypeptides; carboxylic acids and esters; polyols and other; sugar acids and their compounds; sugar derivatives; sugars.

Furthermore, carrying out tests considering two wavelengths of light allows us to draw more specific conclusions. This is because absorbances for those wavelengths depend on different factors: 750 nm stands for optical density, and 590 nm for substrate usage [67]. Based on this knowledge it is possible to calculate the ratio between them [68]. The authors suggested that different ratios might inform varied metabolism responses of examined organisms. According to this research, the lower the ratio the more efficient the metabolism is, and the higher the ratio, the more stressful situation is for the microorganisms [68,69]. Even though the mentioned research was carried out for fungi, this method seems so interesting and universal that we decided to use this approach for research on bacteria.

Table 4 presents the division of chemical compounds present in the GEN III plate (Biolog Inc., Hayward, CA, USA) into the different groups. Moreover, there are 23 chemical resistance assays composed of different pH and salinity levels, antibiotics, and chemical compounds (Table 5). For the most part, a single colony was chosen and mixed in with the Biolog^®^ inoculating fluid IF-A (Biolog Inc., Hayward, CA, USA) to make a solution of the desired turbidity 90–98%. After that, each bacterial suspension was poured into the multichannel pipette reservoir and all wells were filled with 100 μL of prepared suspension. For each isolate there were 3 biological replicates as separate microplates to ensure adequate statistical evidence. The plates were incubated at 28 °C for 72 h in aerobic conditions. The microplates were read manually using the MicroStation (Biolog Inc., Hayward, CA, USA) semiautomated reader at 0, 4, 8, 20, 24, 28, 44, 48, 52, 68, and 72 h for two different wavelengths at 590 nm and 750 nm. The average absorbance for all wells within each category was calculated.

Afterward, the results were exported and subjected to further analysis such as cluster analysis, using Statistica 13.1 software (StatSoft Inc., Tulsa, OK, USA). This analysis was essential for identifying the differences and similarities between the microorganisms used in this study. Furthermore, Sneath’s criteria were applied to hierarchical clustering to find similar groups. Based on the analysis conducted using the metabolic capacity and chemical sensitivity, as well as on antagonistic potential of selected bacteria presented in the first part of the research, there were 3 isolates selected as the best potential candidates to be used in at future biopreparation. Those isolates were *Rhodococcus* sp. B12/18, *Pseudomonas* sp. B37/18, *Arthrobacter* sp. B58/18 and were subjected to additional metabolic and enzymatic analyses using API tests (bioMérieux SA, Marcy l’Etoile, France) and microbiology test plates.

### 4.4. Enzymatic Activity of Selected Bacterial Isolates

Enzymatic activities were evaluated using API ^®^ ZYM strips (bioMérieux SA, Marcy l’Etoile, France). This analysis allows the researcher to detect the presence of 19 different enzymes secreted by the tested microorganisms. Organic farming uses numerous ways of adding exogenous organic matter to soil including organic waste application. Selecting isolates presenting numerous enzymatic activities increases their chance to not only survive after application but also to enhance soil quality utilizing compounds from organic fertilizer making them more accessible to plants. In order to prepare this test, a bacterial inoculum was prepared to achieve the desired opacity equivalent to that of a McFarland No. 5–6 standard. Then, 5 mL of distilled water was added to the incubation chamber to ensure adequate humidity. 

The 65 µL volume of the prepared bacterial solution was added to each measuring cell. Trays with measuring cells were placed inside plastic incubation trays, these in turn were placed in an incubator at 30 °C for 4 h. After this time 5 µL of ZYM A reagent and 5 µL of ZYM B reagents were added to each measuring cell. The strips were set aside for 5 min for the colour to develop. Next, values ranging from ‘−’ to ‘+++’ were assigned, corresponding to the colours developed: ‘−‘ corresponded to a negative reaction, ‘+++’ corresponds to a reaction of maximum intensity and the values ‘+’ or ‘++’ are intermediate reactions depending on the level of intensity. All procedures were performed accordingly to the manufacturer’s manual (bioMérieux SA, Marcy l’Etoile, France).

Other enzymatic activities, including denitrification and nitrification, ammonification, amylolytic, proteolytic, and cellulolytic abilities, nitrogen fixation, and solubilizing phosphate, were determined using common microbiological procedures on Petri dishes with properly selected media. For solid agar media on Petri dish, the plates were inoculated by a loop of bacteria placed at the middle of sterile Petri dish with an appropriate medium. Liquid media were inoculated with a 10% addition of 70% T bacterial solution. Degradation zones for solid media and absorbances for liquid media were measured after 24 and 168 h of incubation.

The proteolytic activity was evaluated using two different media containing nutrient agar and one with the 4% addition of skimmed milk and a second with a 4% gelatin. For the medium that contained gelatin, the degradation zones were measured after covering the plates with Frazier’s reagent.

The amylolytic abilities were evaluated using nutrient agar medium with the addition of 1% starch and the degradation zones were measured after covering the plates with Lugol solution.

The ammonification abilities were evaluated using two different nutrient broths, one containing a 4% addition of skimmed milk and the second one containing 4% addition of urea. A filter-sterilized urea suspension was added to the medium after sterilization to ensure that it has not degraded during sterilization. The absorbance values were determined after the addition of Nessler’s reagent at a wavelength of 410 nm.

Denitrification abilities were determined using a nutrient broth medium with 0.1% addition of KNO_3_. Durham tubes were placed in the test tubes with the medium and bacterial inoculum, and the production of gas was evaluated during a 1-week incubation. Nitrogen fixation abilities were evaluated using a liquid medium without nitrogen source according to the handbook of microbiological media [70]. The absorbance for the wavelength of 600 nm was measured after 24 h and the full incubation period.

The ability of the bacteria to degrade cellulose was evaluated by growing bacteria in 50 mL flasks containing 0.5 g of sterile shredded straw and 2 mL of water. The flasks were inoculated with 1.5 mL of 70% transmittance bacterial solution. After 24 and 168 incubation hours the liquid from the bottom of the flask was transferred to clean test tubes and a reaction with 3,5-dinitrosalicylic acid (DNS) was performed. The absorbance was measured for a wavelength of 550 nm.

In order to evaluate ability to solubilize phosphate, bacteria were inoculated to Pikovskaya agar medium according to the Handbook of Microbiological Media [70]. After 24 and 168 incubation hours, the clean medium degradation zones were measured.

The evaluation of nitrification abilities was performed using the universal mineral medium containing: (NH_4_)_2_SO_4_ 2 g/L, K_2_HPO_4_ 1 g/L, MgSO_4_ 0.5 g/L, NaCl 0.2 g/L, FeSO_4_ 5 mg/L, MnSO_4_ 5 mg/L, CaCO_3_ 5 g/L. Then, 100 mL flasks with 30 mL of sterile medium were inoculated with 1 mL of 70% transmittance bacterial suspension. In order to confirm the presence of NO_2_^−^ ions 1 mL of bacterial culture was added to a well of a 24-well plate. Next, 2 drops of sulphanilic acid and 2 drops of 1-naphthylamine were added to the well. The presence of a pink or brown colour suggests a positive result for the first phase of nitrification. In order to confirm the presence of NO_3_^−^ ions 1 mL of bacterial culture was added to a well of a 24-well plate. Next, 2 drops of diphenylamine were added to the well. The presence of blue or dark blue colour suggests the positive result for the second phase of nitrification. Next, a value ranging from ‘−’ to ‘+++’ was assigned, corresponding to the colours developed: ‘−‘ corresponded to a negative reaction, ‘+++’ to a reaction of maximum intensity and values of ‘+’ or ‘++’ are intermediate reactions depending on the level of intensity.

### 4.5. Metabolic Abilities of Selected Fungal and Fungal-Like Plant Pathogens

Metabolic abilities of selected phytopathogens were performed on the FF MicroPlate (Biolog^®^, Hayward, CA, USA) containing 95 different carbon sources in the wells. Inoculation was performed according to the manufacturer’s protocol with modifications described by Oszust et al. [17]. After the homogenization of the mycelium suspension in inoculating fluid (FF-IF, Biolog^®^, Hayward, CA, USA), the transmittance was adjusted to 75% using a turbidimeter (Biolog^®^, Hayward, CA, USA). A volume of 100 μL of the mycelium suspension was added to each well. The inoculated microplates were incubated in darkness at 25 °C for 10 days. Absorbances were measured daily at the wavelengths of 490 nm and 750 nm, then the ratio of those values was calculated.

### 4.6. Prebiotic Supplement Blend as Possible Component of Future Biopreparation

After GEN III and FF microplate analysis, compounds common for both types of the microplates were chosen. For those substrates, a ratio of absorbance values was calculated, the OD590/OD750 for GEN III and the OD490/OD750 for FF microplates. The ratio was calculated based on the average value from all days of the incubation. Based on the ratio it was possible to indicate some compounds that were causing a stressful metabolic situation to the fungal and fungal-like plant pathogens but were not inhibiting bacterial growth. Those substrates might be considered as a valuable source of additional nutrients for bacterial isolates in biopreparation, thereby further inhibiting growth of pathogenic fungi and/or enhancing beneficial bacterial consortia.

## 5. Conclusions

- Bacteria isolated from raspberry rhizosphere have antagonistic properties against common fungal and fungal-like plant pathogens such as *Botrytis* spp., *Colletotrichum* spp., *Phytophthora* spp., and *Verticillium* spp.
- Describing the utilization abilities of different substrates by microorganisms such as bacteria is a study that might benefit from the application of a calculation method of the 590–750 nm ratio of absorbances. This may not only give the researchers a broader spectrum of results, but also emphasize differences between the tested isolates as far as functional response is concerned, namely the metabolic activity in juxtaposition with biomass production.
- Bacteria to be used in biopreparation need to present some desired features. They should be antagonistic against pathogens, have a broad spectrum of utilized compounds to be used as a carbon source, survive in many different conditions such as different pH values. Due to the fact, that since resistance to antibiotics becomes more common in soil microorganisms it is worth considering that bacteria used in biopreparations are not resistant to antibiotic compounds. This practice reduces spreading this undesirable feature among other microorganisms. From tested bacteria, isolates B12/18 and B58/18 presented needed features and at the same time, they were not resistant to most tested antibiotics. Those two isolates might be worth to be used in future testing and formulations. It is worth considering using other isolates in some special cases and under restrictions due to their good antagonistic and metabolic properties.
- A carbon substrate, such as D-trehalose was utilized by the tested bacteria in a balanced way, namely without causing a stressful metabolic situation that, might be a beneficial addition to a bacterial formulation providing enhanced growth and survivability.
- Metabolic and enzymatic abilities analysis provides important information about particular environmental isolates which might be essential to achieving a complete understanding of their functioning in biopreparations or in future environmental niches. The more enzymatic abilities are demonstrated by the isolates, the easier it might be for those isolates to acclimatize to new environments or stress conditions.
- Carefully selected chemical compounds are a valuable additive to biopreparations. Additives such as D-malic acid, D-saccharic acid, N-acetylo-D-galactosamine, and α-keto-glutaric acid may not only enhance or stimulate bacterial growth but also inhibit the growth of plant fungal and fungal-like pathogens.
- Future research will focus on in planta testing bacterial formulations on raspberry plants combined with introducing fungal and fungal-like pathogens to the experimental treatments. Due to the fact that microbial inoculation may cause tremendous changes in the dynamics of soil microbial communities, subsequent research should include the soil microbiome and mycobiome status and its functional shifts after selected bacterial isolates application. Moreover, regarding the suggested blend of prebiotic supplements, it should be tested for its effect on the growth of beneficial microorganisms and pathogens. Moreover, the study should focus on the development of a biopreparation with carriers and technology conditions appropriate for the selected bacterial isolates, considering future needs arising from the Biodiversity Strategy for 2030.

## Figures and Tables

**Table 1:** Antagonistic properties of bacteria against 4 chosen fungal and fungal-like plants pathogenic isolates. Paper circles inoculated with bacterial inoculum were placed on a PDA plates inoculated with plants pathogen. Pathogen growth inhibition zones were evaluated after 96 h. For each pathogenic fungus, the size of the inhibition zone was determined individually as mean values. For *Colletotrichum* spp. it was <10 mm, 10–15 mm, >15 mm,, for +, ++, +++ respectively, for *Verticillium* spp. it was <10 mm, 10–20 mm, >20 mm, for +, ++, +++ respectively, for *Phytophthora* spp. it was <20 mm, 20–30 mm, >30 mm for +, ++, +++ respectively, for *Botrytis* spp. it was <10 mm, 10–20 mm, >20 mm, for +, ++, +++ respectively. – - means phytopathogen growth without inhibition.

Examined Isolates of Bacteria		Phytopathogens											
		*Colletotrichum* spp.			*Verticillium* spp.			*Phytophthora* spp.			*Botrytis* spp.		
		G172/18	G371/18	G166/18	G293/18	G296/18	G297/18	G368/18	G373/18	G369/18	G275/16	G277/18	G276/18
*Rhodococcus* sp.	B12/18	+	+	++	+	++	++	+++	+	++	−	++	+++
*Pseudomonas* sp.	B25/18	++	++	+++	+++	++	++	++	+	+++	−	−	−
*Pseudomonas* sp.	B37/18	−	−	++	+++	+++	++	+++	+	++	−	−	−
*Bacillus* sp.	B39/18	−	++	++	++	++	++	++	+	+++	++	−	++
*Bacillus* sp.	B40/18	−	−	−	++	+	++	+	+	+	−	−	−
*Arthrobacter* sp.	B49/18	−	−	++	++	++	++	+++	+	++	−	+++	−
*Arthrobacter* sp.	B58/18	++	++	++	++	+	+++	+	+	−	−	++	−
*Rhodococcus* sp.	B73/18	−	−	−	++	++	++	++	+	++	−	++	−

**Table 2:** The OD590/OD750 ratio of the categorized substrates located on Biolog^®^ GEN III microplates. A ratio of >1 indicates a stressful metabolic situation for bacterial isolate functioning. A ratio value of <1 suggests good utilization of substrate (balance between respiration and biomass formation). A ratio value over 1 suggests a stressful metabolic situation, *n* = 3.

Isolate Names	Isolate Number	Amino Acids, Peptides and Polypeptides	Carboxylic Acids and Esters	Polyols	Sugar Acids and Their Compounds	Sugar Derivatives	Sugars
*Arthrobacter* sp.	B49/18	0.54	0.84	0.76	0.00	1.43	0.54
*Arthrobacter* sp.	B58/18	2.15	4.76	1.44	3.19	1.47	2.31
*Bacillus* sp.	B39/18	2.45	2.31	2.18	0.77	1.66	0.85
*Bacillus* sp.	B40/18	2.26	2.01	2.19	0.76	1.14	0.56
*Pseudomonas* sp.	B25/18	1.81	1.49	1.79	0.58	1.26	0.51
*Pseudomonas* sp.	B37/18	2.19	2.17	2.41	0.80	1.39	0.62
*Rhodococcus* sp.	B73/18	2.44	2.20	1.91	0.77	1.32	0.61
*Rhodococcus* sp.	B12/18	1.28	1.41	0.89	0.25	1.75	0.81

**Table 3:** Enzymatic abilities of selected beneficial bacterial isolates. ‘-‘ corresponds to a negative reaction, ‘+++’ to a reaction of maximum intensity and values ‘+’ or ‘++’ are intermediate reactions depending on the level of intensity, *n* = 3.

Metabolic Abilities	Substrate Additive	*Rhodococcus* sp. B12/18		*Pseudomonas* sp. B37/18		*Arthrobacter* sp. B58/18	
		24 h	168 h	24 h	168 h	24 h	168 h
Proteolytic	4% skim milk	−	−	+	+++	−	−
	4% gelatin	−	−	+++	+++	−	+
Amylolytic	1% starch	−	−	++	+++	+	++
Amonification	4% skim milk	+	+	+++	+++	++	++
	4% urea	++	++	−	−	+	+
Denitryfication	0.1% KNO_3_	+	+	−	−	+	++
Nitrogen fixation	Medium without nitrogen	−	−	−	−	−	−
Cellulolytic	Shredded straw	−	+	+	+++	++	+++
Nitryfication	Nitryfication medium	−	−	−	−	−	−
Phosphate solubilization	Pikovska medium	−	−	−	−	−	−
**Enzymatic activity**	**Substrate**	***Rhodococcus* sp. B12/18**		***Pseudomonas* sp. B37/18**		***Arthrobacter* sp. B58/18**	
Alkaline phosphatase	2-naphthyl phosphate	++		−		−	
Esterase (C 4)	2-naphthyl butyrate	++		++		+	
Lipase esterase (C 8)	2-naphthyl caprylate	+++		+		+	
Lipase (C 14)	2-naphthyl myristate	−		−		+	
Leucine arrylamidase	L-leucyl-2-naphthyllamide	+++		++		+++	
Valine arrylamidase	L-valyl-2-naphthlamide	++		−		++	
Cystine arrylamidase	L-cystyl-2-naphthlamide	+		−		++	
Trypsin	N-benzoyl-DL-arginine-2-naphthyllamide	−		−		−	
α-chymotrypsin	N-glutaryl-phenylalanine-2-naphthyllamide	+		−		−	
Acid phosphatase	2-naphthyl phosphate	++		+		+	
Naphthyl-AS-BI phosphohydrolase	Naphthyl AS-BI-phosphate	+++		++		++	
α-galactosidase	6-Br-2-naphthyl-αD-galactopyranoside	−		−		++	
ß-galactosidase	2-naphthyl-ßD-galactopyranoside	−		−		−	
ß-glucuronidase	Naphthyl-AS-BI-ßD-glucuronide	−		−		−	
α-glucosidase	2-naphthyl-αD-glucopyranoside	+++		−		++	
ß-glucosidase	6-Br-2-naphthyl-ßD-glucopyranoside	+++		−		−	
N-acetyl-ß-glucosaminidase	1-naphthyl-N-acetyl-ßD-glucosaminide	−		−		−	
α-mannosidase	6-Br-2-naphthyl-αD-mannopyranoside	−		−		+	
α-fucosidase	2-naphthyl-αL-fucopyranoside	−		−		−	

**Table 4:** The assignment of individual carbon sources located in the GEN III plates (Biolog Inc., Hayward, CA, USA) into categorized groups.

Amino Acids, Peptideds and Polypeptides	Polyols and Others	Sugars	Sugar Acids and Their Compounds	Carboxylic Acids and Esters	Sugar Derivatives
D-Aspartic Acid	D-Sorbitol	Dextrin	D-Galacturonic Acid	p-Hydroxyphenylacetic Acid	β-Methyl-D-glucoside
D-Serine	D-Mannitol	D-Maltose	L-Galactonic Acid lactone	L-Lactic Acid	D-Salicin
Glycyl-L-proline	D-Arabitol	D-Trehalose	D-Gluconic Acid	Citric Acid	N-Acetyl-D-glucosamine
L-Alanine	Myo-inositol	D-Cellobiose	D-Glucuronic Acid	α-Ketoglutaric Acid	N-Acetyl-β-D-mannosamine
L-Arginine	Glycerol	Gentiobiose	Glucuronamide	D-Malic Acid	N-Acetyl-D-galactosamine
L-Aspartic Acid	Tween 40	Sucrose	Mucic Acid	L-Malic Acid	Inosine
L-Glutamic Acid		D-Turanose	Quinic Acid	Bromosuccinic Acid	D-Glucose-6-PO_4_
L-Histidine		Stachyose	D-Saccharic Acid	α-Hydroxybutyric Acid	D-Fructose-6-PO_4_
L-Pyroglutamic Acid		D-Raffinose		β-Hydroxy-D	
L-Serine		α-D-Lactose		α-Ketobutyric Acid	
γ-Amino-butyric Acid		D-Melibiose		Acetoacetic Acid	
Gelatin		N-Acetylneuraminic Acid		Propionic Acid	
		α-D-Glucose		Acetic Acid	
		D-Mannose		Formic Acid	
		D-Fructose		Methyl Pyruvate	
		D-Galactose		D-Lactic Acid Methyl Ester	
		3-Methylglucose		L-Butyric Acid	
		D-Fucose			
		L-Fucose			
		L-Rhamnose			
		Pectin			

**Table 5:** The assignment of individual chemical compounds located in the GEN III plates (Biolog Inc., Hayward, CA, USA) into categorized groups.

Antibiotics	Organic Compounds	Chemical Soil Properties		Toxic Substances
		pH	Salinity	
Fusidic Acid	1% Sodium Lactate	pH 6	1% NaCl	Guanidine HCl
Troleandomycin	D-serine	pH 5	4% NaCl	Niaproof 4
Rifamycin SV	Sodium Butyrate		8% NaCl	Tetrazolium Violet
Minocycline	Sodium Bromate			Tetrazolium Blue
Lincomycin				Lithium Chloride
Vancomycin				Potassium tellurite
Nalidixic Acid				
Aztreonam				

## Abbreviations

16S rDNA	16S ribosomal DNA gene
ASM	Acibenzolar-S-methyl
MEGA	Molecular Evolutionary Genetics Analysis
OD590/OD750 OD490/OD750	The ratio of absorbances value measured at 590 nm and 750 nm The ratio of absorbances value measured at 490 nm and 750 nm
PCA	Plate Count Agar
PCR	Polymerase Chain Reaction
PDA	Potato Dextrose Agar
T	Transmittance

## Appendix A

**Table A1:** Antagonistic properties of all bacteria isolated from soil and potentially beneficial bacteria against 4 selected plant fungal pathogens. Paper circles inoculated with bacterial inoculum were placed on a PDA plate inoculated with plant fungal pathogen. Pathogen growth inhibition zones were evaluated after 96 incubation hours. Pluses and minuses represent the size of an inhibition zone. For each pathogens the size of the inhibition zone was determined individually as mean values. For *Colletotrichum* spp. it was 18 mm ± 2.6, 22 mm ± 10.2, 30.6 mm ± 6.1, for +, ++, +++ respectively, for *Verticillium* spp. it was 13.0 mm ± 5.0, 14.9 mm ± 2.7, 18.2 mm ± 2.5, for +, ++, +++ respectively, for *Phytophthora* spp. it was 18.0 mm ± 7.6, 23.3 mm ± 2.5, 45.0 mm ± 6.5 for +, ++, +++ respectively, for *Botrytis* spp. it was 14.4 mm ± 1.9, 17.6 mm ± 2.7, 26.1 mm ± 3.1 for +, ++, +++ respectively. – means good growth of phytopathogens without inhibition effect.

Bacteria		Phytopathogenic Fungi and Fungal-Like Pathogens											
		*Colletotrichum* spp.			*Verticillium* spp.			*Phytophthora* spp.			*Botrytis* spp.		
		G172/18	G371/18	G166/18	G293/18	G296/18	G297/18	G368/18	G373/18	G369/18	G275/16	G277/18	G276/18
*Flavobacterium* sp.	B10/18	−	−	−	−	−	−	−	−	−	-	−	−
*Rhodococcus* sp.	B12/18	+	+	++	+	++	++	+++	+	++	-	++	+++
*Pseudomonas* sp.	B13/18	−	−	−	−	−	−	−	−	−	−	−	−
*Pseudomonas* sp.	B14/18	−	−	−	−	−	−	−	−	+	−	++	−
*Burkholderia* sp.	B18/18	−	−	−	−	−	−	−	−	−	−	−	−
*Curtobacterium* sp.	B19/18	−	−	−	−	−	−	−	−	−	−	−	−
*Arthrobacter* sp.	B20/18	−	−	−	−	−	−	−	−	−	−	+	−
*Pseudomonas* sp.	B21/18	−	−	−	−	−	−	−	+	−	−	−	+
*Pseudomonas* sp.	B22/18	−	−	−	−	−	−	−	−	−	−	−	−
*Microbacterium* sp.	B23/18	−	−	−	−	−	−	−	++	-	-	-	−-
*Pseudomonas* sp.	B25/18	++	++	+++	+++	++	++	++	+	+++	−	−	−
*Pseudomonas* sp.	B26/18	−	−	−	−	−	−	−	−	−	−	−	−
*Bacillus* sp.	B27/18	−	−	−	−	+	+	−	−	−	−	−	−
not identified	B28/18	−	−	−	−	−	−	−	+	−	−	−	−
*Pseudomonas* sp.	B29/18	−	−	−	++	+	−	−	−	−	−	−	−
*Pseudomonas* sp.	B30/18	−	−	+	+	+	−	−	+	−	−	−	−
*Bacillus* sp.	B31/18	−	−	−	−	−	−	−	−	−	−	−	−
*Bacillus* sp.	B32/18	−	−	−	−	−	+	−	−	−	−	−	−
*Pseudomonas* sp.	B33/18	+	−	−	+	+	−	−	++	−	−	−	−
*Arthrobacter* sp.	B35/18	−	−	−	−	−	−	−	++	−	−	−	−
*Pseudomonas* sp.	B37/18	−	−	++	+++	+++	++	+++	+	++		−	
*Bacillus* sp.	B39/18	−	++	++	++	++	++	++	+	+++	++	−	++
*Bacillus* sp.	B40/18	−	−	−	++	+	++	+	+	+	−	−	−
*Flavobacterium* sp.	B41/18	−	−	−	−	−	−	+	−	−	−	−	−
*Pseudomonas* sp.	B42/18	−	−	−	−	−	−	−	++	+	−	−	−
*Pedobacter* sp.	B44/18	−	−	−	−	−	+	++	++	+	−	−	−
*Arthrobacter* sp.	B45/18	−	−	−	−	−	−	−	++	+	+	−	−
*Pseudomonas* sp.	B46/18	-	-	-	-	+	+	-	+	-	-	−	−
*Plantibacter* sp.	B47/18	−	−	−	−	−	−	−	−	−	−	−	−
*Pseudomonas* sp.	B48/18	−	−	−	−	−	−	−	−	++	−	−	−
*Arthrobacter* sp.	B49/18	-	-	++	++	++	++	+++	+	++	-	+++	-
*Pedobacter* sp.	B51/18	−	−	−	−	−	−	−	−	−	−	−	−
*Janthinobacterium* sp.	B54/18	−	−	−	−	−	−	−	−	−	−	−	−
*Novosphingobium* sp.	B57/18	−	−	−	−	++	++	−	+++	−	−	−	−
*Arthrobacter* sp.	B58/18	++	++	++	++	+	+++	+	+	−	−	++	−
*Arthrobacter* sp.	B59/18	−	−	−	−	−	−	−	−	++	−	−	−
not identidied	B60/18	−	−	−	−	+	+	++	−	−	−	−	−
*Bacillus* sp.	B61/18	−	−	−	−	−	−	−	−	−	−	−	−
*Flavobacterium* sp.	B66/18	−	−	−	++	+	++	−	+++	−	−	−	−
*Bacillus* sp.	B68/18	−	−	−	−	+	−	−	-	−	−	−	−
*Bacillus* sp.	B69/18	−	−	−	++	-	−	−	+++	−	−	−	−
*Shinella* sp.	B70/18	−	−	−	−	−	−	−	+++	−	−	−	−
*Microbacterium* sp.	B72/18	−	−	−	−	−	−	++	+	+	−	−	−
*Rhodococcus* sp.	B73/18	-	-	-	++	++	++	++	+	++	−	++	−
*Arthrobacter* sp.	B74/18	+	−	++	+	−	−	−	++	−	−	−	−
*Pseudomonas* sp.	B75/18	−	+++	−	+	−	−	−	+++	+	−	++	−
*Bacillus* sp.	B77/18	−	−	−	−	+	+	++	+	−	−	+	−

**Table A2:** The table presents values of the ratio calculated based on absorbance values the OD590/OD750 for beneficial bacterial isolates and the OD490/OD750 nm for plant fungal and fungal-like pathogens. Ratios were calculated from average absorbance values from all measurements. Values higher than >1 suggest stressful metabolic situation, values lover than <1 suggest good utilization of substrates. Underlined substrates are those that are neutral for bacteria, but cause stressful metabolic situation for plant fungal and fungal-like pathogens.

Substrate	Beneficial Bacteria			Fungal and Fungal-Like Plant Pathogens									
	*Rhodococcus* sp.	*Pseudomonas* sp.	*Arthrobacter* sp.	*Botrytis* spp.			*Colletotrichum* spp.			*Phytophthora* spp.	*Verticillium* spp.		
	B12/18	B37/18	B58/18	G275/18	G276/18	G277/18	G166/18	G172/18	G371/18	G408/18	G293/18	G296/18	G297/18
Bromosuccinic Acid	0.00	0.16	8.60	2.05	2.91	3.84	17.67	0.00	11.44	0.00	13.91	8.50	12.38
D-Arabitol	0.00	0.90	8.37	1.23	1.60	1.49	1.19	1.53	1.29	1.86	6.82	3.48	3.42
D-Cellobiose	0.00	0.00	3.17	1.55	1.44	1.20	1.81	1.65	1.16	1.24	2.53	2.05	2.42
Dextrin	9.32	0.00	1.56	0.00	1.85	4.14	2.38	2.98	1.74	1.93	2.27	2.08	2.31
D-Fructose	22.84	0.00	18.46	1.39	1.42	1.24	1.80	1.53	1.73	1.30	1.64	1.61	1.67
D-Galactose	0.00	0.00	0.00	1.38	1.42	1.37	1.35	1.59	1.31	1.39	1.61	1.59	1.65
D-Galacturonic Acid	0.00	0.00	16.97	1.65	1.95	1.43	2.49	2.03	1.78	1.64	0.00	0.00	0.00
D-Glucuronic Acid	0.00	0.00	30.14	1.54	2.16	49.57	1.73	3.40	2.35	7.11	2.18	1.99	2.04
D-Lactic Acid Methyl Ester	0.00	0.00	9.94	0.00	0.00	0.00	0.00	0.00	0.00	0.00	0.00	0.00	0.00
**D-Malic Acid**	**0.00**	**0.00**	**7.43**	**2.70**	**2.92**	**4.66**	**4.80**	**5.24**	**2.59**	**6.13**	**2.09**	**2.07**	**2.11**
D-Mannitol	2.31	24.86	19.51	1.36	1.42	1.40	1.18	1.42	1.33	1.30	2.44	2.23	3.00
D-Mannose	3.30	3.76	7.48	1.62	1.54	1.34	3.28	1.90	1.55	1.31	1.73	1.66	1.80
D-Melibiose	0.00	0.00	0.00	1.70	1.43	1.43	1.34	1.55	1.21	1.41	1.46	1.38	1.51
D-Raffinose	6.03	0.00	2.70	1.40	1.45	1.34	1.24	1.39	1.33	1.23	1.52	1.51	1.52
**D-Saccharic Acid**	**0.00**	**0.00**	**54.50**	**1.69**	**2.76**	**7.70**	**2.04**	**8.50**	**2.57**	**0.00**	**3.58**	**3.63**	**2.64**
D-Sorbitol	19.00	0.00	0.00	1.38	1.36	1.20	1.46	1.93	1.41	1.22	6.71	7.47	6.36
D-Trehalose	3.76	0.00	4.15	1.76	1.43	1.54	1.76	1.30	1.35	1.71	1.52	1.56	1.54
Gentiobiose	68.20	0.00	9.61	1.56	1.50	1.46	2.40	1.65	1.41	1.23	1.68	1.57	1.70
Glucuronamide	0.00	0.00	17.05	0.00	0.00	3.50	0.00	0.00	0.00	0.00	0.00	0.00	0.00
Glycerol	2.50	6.47	0.00	1.39	1.63	1.27	1.66	1.57	1.63	1.33	1.70	1.97	1.96
L-Alanine	3.82	1.17	19.17	2.26	2.19	1.89	1.48	1.77	1.43	1.78	2.10	2.42	2.59
L-Aspartic Acid	3.44	2.93	24.17	3.09	5.22	4.05	2.85	3.28	2.69	0.00	3.35	3.70	3.56
L-Fucose	0.00	0.00	127.57	0.00	0.00	0.00	0.00	0.00	0.00	0.00	2.70	2.33	3.91
L-Glutamic Acid	0.00	2.46	9.13	2.27	2.42	2.00	2.94	5.13	1.63	3.34	3.23	2.46	3.97
L-Lactic Acid	3.42	3.41	0.00	0.00	0.00	0.00	0.00	0.00	13.15	0.00	255.50	5.43	14.76
L-Malic Acid	3.01	30.46	8.28	0.00	4.90	0.00	6.63	5.89	2.83	0.00	3.94	3.92	4.49
L-Pyroglutamic Acid	2.34	5.90	0.00	0.00	0.00	0.00	2.19	1.82	1.80	0.00	0.00	0.00	0.00
L-Rhamnose	0.00	0.00	82.45	1.27	1.47	1.33	1.26	1.18	1.25	1.20	1.53	1.51	1.58
L-Serine	3.30	0.52	6.45	1.92	24.50	3.03	2.28	2.96	1.90	6.94	2.58	2.54	2.82
**N-Acetyl-D-Galactosamine**	**0.00**	**0.00**	**15.67**	**1.50**	**1.73**	**3.20**	**1.16**	**1.40**	**1.23**	**1.49**	**1.56**	**1.78**	**1.70**
N-Acetyl-D-Glucosamine	2.23	0.00	3.99	0.00	0.00	0.00	0.00	0.00	0.00	0.00	0.00	0.00	0.00
N-Acetyl-D-Mannosamine	0.00	0.00	6.17	0.00	0.00	0.00	0.00	0.00	0.00	0.00	0.00	0.00	0.00
Quinic Acid	2.26	0.00	11.40	1.57	1.94	1.74	1.41	1.70	1.30	2.57	3.61	2.65	2.87
Stachyose	21.00	0.00	5.45	1.37	1.33	1.43	1.33	1.62	1.26	1.34	1.46	1.46	1.50
Sucrose	9.46	0.00	3.71	1.45	1.40	1.46	1.68	1.53	1.73	1.32	1.54	1.55	1.56
α-D-Glucose	1.51	4.07	8.24	1.56	1.40	1.41	1.70	1.33	1.18	1.46	1.62	1.62	1.68
α-D-Lactose	139.50	0.00	13.03	1.42	1.39	1.33	1.46	1.83	1.41	1.26	0.00	0.00	0.00
**α-Keto-glutaric Acid**	**5.97**	**0.00**	**0.00**	**78.29**	**5.07**	**0.00**	**3.80**	**5.58**	**8.88**	**21.06**	**8.26**	**5.57**	**6.92**
β-Methyl-D-Glucoside	38.80	0.00	7.34	1.79	1.51	1.73	1.49	1.68	1.44	1.45	1.52	1.44	1.54
γ-Amino-butyric Acid	2.00	0.00	1.74	1.41	7.05	3.19	1.39	1.38	1.43	2.17	2.11	2.30	2.50

**Table A3:** Data presenting information about all isolates including identification, accession number of sequences in GenBank, the sample from which isolation was made, the medium used for isolation, and forest, where samples were obtained. LMEM – Laboratory of Molecular and Environmental Microbiology of the Institute of Agrophysics, Polish Academy of Sciences.

Isolate Code LMEM	Identification	The Accession Number of Sequences in GenBank	Compartment	Microbiological Medium	Forest Location in Poland	Coordinates	Forest Distinct in Poland
B10/18	*Flavobacterium* sp.	MW255682	wild raspberry rhizosphere	Plate Count Agar	Chruślanki Józefowskie	N 51.01061 E 022.01705	Kraśnik
B11/18	*Arthrobacter* sp.	MW255683	wild raspberry rhizosphere	Plate Count Agar	Chruślanki Józefowskie	N 51.01061 E 022.01705	Kraśnik
B12/18	*Rhodococcus* sp.	MW255650	wild raspberry rhizosphere	Agar with soil extract	Chruślanki Józefowskie	N 51.01061 E 022.01705	Kraśnik
B13/18	*Pseudomonas* sp.	MW255684	wild raspberry rhizosphere	Agar with soil extract	Chruślanki Józefowskie	N 51.01061 E 022.01705	Kraśnik
B14/18	*Pseudomonas* sp.	MW255685	wild raspberry rhizosphere	Agar with soil extract	Chruślanki Józefowskie	N 51.01061 E 022.01705	Kraśnik
B15/18	*Pseudomonas* sp.	MW255686	wild raspberry rhizosphere	Plate Count Agar	Pomorze	N 51.17137 E 021.95993	Kraśnik
B16/18	*Arthrobacter* sp.	MW255687	wild raspberry rhizosphere	Agar with soil extract	Wierzchowiska	-	Janów Lubelski
B17/18	*Mucilaginibacter* sp.	MW255688	wild raspberry rhizosphere	Agar with soil extract	Wierzchowiska	-	Janów Lubelski
B18/18	*Burkholderia* sp.	MW255689	wild raspberry rhizosphere	Agar with soil extract	Świdnik	N 51.54462 E 022.28306	Świdnik
B19/18	*Curtobacterium* sp.	MW255690	wild raspberry rhizosphere	Plate Count Agar	Chruślanki Józefowskie	N 51.01083 E 022.01578	Kraśnik
B20/18	*Arthrobacter* sp.	MW255691	wild raspberry rhizosphere	Plate Count Agar	Pomorze	N 51.17137 E 021.95993	Kraśnik
B21/18	*Pseudomonas* sp.	-	wild raspberry rhizosphere	Plate Count Agar	Pomorze	N 51.17137 E 021.95993	Kraśnik
B22/18	*Pseudomonas* sp.	MW255692	wild raspberry rhizosphere	Plate Count Agar	Chruślanki Józefowskie	N 51.01061 E 022.01705	Kraśnik
B23/18	*Microbacterium* sp.	MW255693	wild raspberry rhizosphere	Agar with soil extract	Chruślanki Józefowskie	N 51.01051 E 022.01672	Kraśnik
B24/18	*Xanthomonas* sp.	MW255694	wild raspberry rhizosphere	Plate Count Agar	Wierzchowiska	-	Janów Lubelski
B25/18	*Pseudomonas protegens*	MW255695	wild raspberry rhizosphere	Plate Count Agar	Chruślanki Józefowskie	N 51.00903 E 022.01139	Kraśnik
B26/18	*Pseudomonas mohnii*	MW255696	wild raspberry rhizosphere	Plate Count Agar	Pomorze	N 51.17247 E 021.95057	Kraśnik
B27/18	*Bacillus mycoides*	MW255697	wild raspberry rhizosphere	Plate Count Agar	Chruślanki Józefowskie	N 51.00903 E 022.01139	Kraśnik
B29/18	*Pseudomonas* sp.	MW255698	wild raspberry rhizosphere	Plate Count Agar	Chruślanki Józefowskie	N 51.01083 E 022.01578	Kraśnik
B30/18	*Pseudomonas* sp.	MW255699	wild raspberry rhizosphere	Plate Count Agar	Wierzchowiska	-	Janów Lubelski
B31/18	*Bacillus* sp.	MW255700	wild raspberry rhizosphere	Plate Count Agar	Chruślanki Józefowskie	N 51.00936 E 022.01220	Kraśnik
B32/18	*Bacillus mycoides*	MW255701	wild raspberry rhizosphere	Plate Count Agar	Bobowiska	N 51.41395 E 022.24402	Puławy
B33/18	*Pseudomonas* sp.	MW255702	wild raspberry rhizosphere	Plate Count Agar	Bobowiska	N 51.41395 E 022.24402	Puławy
B34/18	*Erwinia* sp.	MW255703	wild raspberry rhizosphere	Plate Count Agar	Krzywda	-	Łuków
B35/18	*Arthrobacter psychrolactophilus*	MW255704	wild raspberry rhizosphere	Plate Count Agar	Krzywda	-	Łuków
B36/18	*Pseudomonas* sp.	MW255705	wild raspberry rhizosphere	Plate Count Agar	Chruślanki Józefowskie	N 51.01061 E 022.01705	Kraśnik
B37/18	*Pseudomonas* sp.	MW255651	wild raspberry rhizosphere	Plate Count Agar	Chruślanki Józefowskie	N 51.01061 E 022.01705	Kraśnik
B38/18	*Leifsonia* sp.	MW255706	wild raspberry rhizosphere	Plate Count Agar	Śmiary	-	Siedlce
B39/18	*Bacillus thuringiensis*	MW255707	wild raspberry rhizosphere	Plate Count Agar	Chruślanki Józefowskie	N 51.01051 E 022.01672	Kraśnik
B40/18	*Bacillus* sp.	MW255708	wild raspberry rhizosphere	Plate Count Agar	Chruślanki Józefowskie	N 51.01061 E 022.01705	Kraśnik
B41/18	*Flavobacterium* sp.	-	wild raspberry rhizosphere	Plate Count Agar	Chruślanki Józefowskie	N 51.00926 E 022.01120	Kraśnik
B42/18	*Pseudomonas* sp.	MW255709	wild raspberry rhizosphere	Plate Count Agar	Bobowiska	N 51.41395 E 022.24402	Puławy
B43/18	*Pedobacter* sp.	-	wild raspberry rhizosphere	Plate Count Agar	Bobowiska	N 51.41395 E 022.24402	Puławy
B44/18	*Pedobacter* sp.	MW255710	wild raspberry rhizosphere	Plate Count Agar	Bobowiska	N 51.41395 E 022.24402	Puławy
B45/18	*Arthrobacter* sp.	MW255711	wild raspberry rhizosphere	Plate Count Agar	Pomorze	N 51.17100 E 021.95998	Kraśnik
B46/18	*Pseudomonas fluorescens*	MW255712	wild raspberry rhizosphere	Plate Count Agar	Pomorze	N 51.17100 E 021.95998	Kraśnik
B47/18	*Plantibacter* sp.	MW255713	wild raspberry rhizosphere	Plate Count Agar	Pomorze	N 51.17100 E 021.95998	Kraśnik
B48/18	*Pseudomonas* sp.	MW255714	wild raspberry rhizosphere	Plate Count Agar	Pomorze	N 51.17100 E 021.95998	Kraśnik
B49/18	Arthrobacter sp.	MW255715	wild raspberry rhizosphere	Plate Count Agar	Bobowiska	N 51.41395 E 022.24402	Puławy
B50/18	*Chryseobacterium balustinum*	MW255716	wild raspberry rhizosphere	Plate Count Agar	Śmiary	-	Siedlce
B51/18	*Pedobacter* sp.	MW255717	wild raspberry rhizosphere	Plate Count Agar	Śmiary	-	Siedlce
B52/18	*Luteibacter rhizovicinus*	MW255718	wild raspberry rhizosphere	Plate Count Agar	Śmiary	-	Siedlce
B53/18	*Burkholderia* sp.	MW255719	wild raspberry rhizosphere	Agar with soil extract	Śmiary	-	Siedlce
B54/18	*Janthinobacterium lividum*	MW255720	wild raspberry rhizosphere	Agar with soil extract	Śmiary	-	Siedlce
B56/18	*Burkholderia* sp.	MW255721	wild raspberry rhizosphere	Agar with soil extract	Chruślanki Józefowskie	N 51.00926 E 022.01120	Kraśnik
B57/18	*Novosphingobium* sp.	MW255722	wild raspberry rhizosphere	Agar with soil extract	Pomorze	N 51.17137 E 021.95993	Kraśnik
B58/18	*Arthrobacter globiformis*	MW255652	wild raspberry rhizosphere	Agar with soil extract	Wierzchowiska	-	Janów Lubelski
B59/18	*Arthrobacter* sp.	-	wild raspberry rhizosphere	Agar with soil extract	Pomorze	N 51.17137 E 021.95993	Kraśnik
B61/18	*Bacillus simplex*	MW255723	wild raspberry rhizosphere	Plate Count Agar	Chruślanki Józefowskie	N 51.01083 E 022.01578	Kraśnik
B62/18	*Chryseobacterium* sp.	MW255724	wild raspberry rhizosphere	Plate Count Agar	Chruślanki Józefowskie	N 51.01083 E 022.01578	Kraśnik
B63/18	*Pseudomonas* sp.	MW255725	wild raspberry rhizosphere	Plate Count Agar	Chruślanki Józefowskie	N 51.01083 E 022.01578	Kraśnik
B64/18	*Variovorax* sp.	MW255726	wild raspberry rhizosphere	Plate Count Agar	Chruślanki Józefowskie	N 51.01083 E 022.01578	Kraśnik
B65/18	*Pseudomonas* sp.	MW255727	wild raspberry rhizosphere	Plate Count Agar	Chruślanki Józefowskie	N 51.00926 E 022.01120	Kraśnik
B66/18	*Flavobacterium* sp.	-	wild raspberry rhizosphere	Plate Count Agar	Chruślanki Józefowskie	N 51.01083 E 022.01578	Kraśnik
B67/18	*Burkholderia* sp.	MW255728	wild raspberry rhizosphere	Plate Count Agar	Chruślanki Józefowskie	N 51.00903 E 022.01139	Kraśnik
B68/18	*Bacillus mycoides*	MW255647	wild raspberry roots	Plate Count Agar	Chruślanki Józefowskie	N 51.00903 E 022.01139	Kraśnik
B69/18	*Bacillus simplex*	MW255648	wild raspberry roots	Plate Count Agar	Chruślanki Józefowskie	N 51.01083 E 022.01578	Kraśnik
B70/18	*Shinella* sp.	MW255729	wild raspberry rhizosphere	Plate Count Agar	Wierzchowiska	-	Janów Lubelski
B71/18	*Roseomonas mucosa*	MW255730	wild raspberry rhizosphere	Plate Count Agar	Chruślanki Józefowskie	N 51.00926 E 022.01120	Kraśnik
B72/18	*Microbacterium* sp.	MW255731	wild raspberry rhizosphere	Plate Count Agar	Śmiary	-	Siedlce
B73/18	*Rhodococcus erythropolis*	MW255732	wild raspberry rhizosphere	with soil extract	Bobowiska	N 51.41395 E 022.24402	Puławy
B74/18	*Arthrobacter* sp.	MW255733	wild raspberry rhizosphere	Plate Count Agar	Pomorze	N 51.17137 E 021.95993	Kraśnik
B75/18	*Arthrobacter* sp.	MW255653	wild raspberry rhizosphere	Plate Count Agar	Chruślanki Józefowskie	N 51.00926 E 022.01120	Kraśnik
B76/18	*Pseudomonas* sp.	MW255734	wild raspberry rhizosphere	Plate Count Agar	Chruślanki Józefowskie	N 51.01083 E 022.01578	Kraśnik
B77/18	*Bacillus* sp.	MW255735	wild raspberry rhizosphere	Plate Count Agar	Wierzchowiska	-	Janów Lubelski
